# Deciphering Gut Microbiota Dysbiosis and Corresponding Genetic and Metabolic Dysregulation in Psoriasis Patients Using Metagenomics Sequencing

**DOI:** 10.3389/fcimb.2021.605825

**Published:** 2021-04-01

**Authors:** Shiju Xiao, Guangzhong Zhang, Chunyan Jiang, Xin Liu, Xiaoxu Wang, Yafan Li, Meijiao Cheng, Hongpeng Lv, Fuyang Xian, Xinwei Guo, Yong Tan

**Affiliations:** ^1^ Beijing Hospital of Traditional Chinese Medicine, Capital Medical University, Beijing, China; ^2^ Graduate School, Capital Medical University, Beijing, China; ^3^ Puyang Hospital of Traditional Chinese Medicine, Puyang, China; ^4^ Beijing University of Chinese Medicine, Beijing, China; ^5^ Beijing QuantiHealth Technology Co., Ltd, Beijing, China; ^6^ Beijing Institute of Traditional Chinese Medicine, Beijing, China; ^7^ Institute of Basic Research in Clinical Medicine, China Academy of Chinese Medical Sciences, Beijing, China

**Keywords:** gut microbiota, genetic functions, metabolites, psoriasis, metagenomics sequencing

## Abstract

**Background:**

Increasing evidence has shown that alterations in the intestinal microbiota play an important role in the pathogenesis of psoriasis. The existing relevant studies focus on 16S rRNA gene sequencing, but in-depth research on gene functions and comprehensive identification of microbiota is lacking.

**Objectives:**

To comprehensively identify characteristic gut microbial compositions, genetic functions and relative metabolites of patients with psoriasis and to reveal the potential pathogenesis of psoriasis.

**Methods:**

DNA was extracted from the faecal microbiota of 30 psoriatic patients and 15 healthy subjects, and metagenomics sequencing and bioinformatic analyses were performed. The Kyoto Encyclopedia of Genes and Genomes (KEGG) database, cluster of orthologous groups (COG) annotations, and metabolic analyses were used to indicate relative target genes and pathways to reveal the pathogenesis of psoriasis.

**Results:**

Compared with healthy individuals, the gut microbiota of psoriasis patients displayed an alteration in microbial taxa distribution, but no significant difference in microbial diversity. A distinct gut microbial composition in patients with psoriasis was observed, with an increased abundance of the phyla *Firmicutes*, *Actinobacteria* and *Verrucomicrobia* and genera *Faecalibacterium, Bacteroides*, *Bifidobacterium*, *Megamonas* and *Roseburia* and a decreased abundance of the phyla *Bacteroidetes, Euryarchaeota* and *Proteobacteria* and genera *Prevotella, Alistipes*, and *Eubacterium*. A total of 134 COGs were predicted with functional analysis, and 15 KEGG pathways, including lipopolysaccharide (LPS) biosynthesis, WNT signaling, apoptosis, bacterial secretion system, and phosphotransferase system, were significantly enriched in psoriasis patients. Five metabolites, hydrogen sulfide (H_2_S), isovalerate, isobutyrate, hyaluronan and hemicellulose, were significantly dysregulated in the psoriatic cohort. The dysbiosis of gut microbiota, enriched pathways and dysregulated metabolites are relevant to immune and inflammatory response, apoptosis, the vascular endothelial growth factor (VEGF) signaling pathway, gut-brain axis and brain-skin axis that play important roles in the pathogenesis of psoriasis.

**Conclusions:**

A clear dysbiosis was displayed in the gut microbiota profile, genetic functions and relative metabolites of psoriasis patients. This study is beneficial for further understanding the inflammatory pathogenesis of psoriasis and could be used to develop microbiome-based predictions and therapeutic approaches.

## Introduction

Psoriasis is an immune-mediated inflammatory chronic skin disease characterized by chronic inflammation in the dermis, parakeratosis and excessive epidermal growth, and 2-3% of the population are affected by psoriasis (Parisi et al., 2013; Hidalgo-Cantabrana et al., 2019). Less attention has been focused on the relationship between intestinal microbiota dysbiosis and the pathogenesis of psoriasis (Yan et al., 2017). However, recent studies have reported an alteration in the gut microbiota in patients with psoriasis (Scher et al., 2015; Chen et al., 2018; Hidalgo-Cantabrana et al., 2019; Shapiro et al., 2019) that is related to an aberrant inflammatory response (Salem et al., 2018). The intestinal microbiota plays an important role in the maintenance of immune homeostasis, regulating both acquired and innate immune responses (Sommer and Backhed, 2013; Palm et al., 2015; Planer et al., 2016). Gut microbiota alterations may activate T cells, trigger an inflammatory process and induce immune disorders (Littman and Pamer, 2011; Collins et al., 2012; Honda and Littman, 2016). Gut microbiota dysbiosis is related to immune and autoimmune disorders such as rheumatoid arthritis (RA) (Yeoh et al., 2013), allergies (Chen et al., 2016), type 1 diabetes (de Groot et al., 2017), autism (Mangiola et al., 2016; Zhang et al., 2018), inflammatory bowel disease (IBD) (Huttenhower et al., 2014), systemic lupus erythematosus (SLE) (Edwards and Costenbader, 2014), and multiple sclerosis (Rosser and Mauri, 2016). Moreover, intestinal microbiota alteration plays a key role in psoriasis (Shamriz et al., 2016; Luo et al., 2017; Stockinger and Omenetti, 2017), as well as other skin alterations, such as atopic dermatitis and vitiligo (Penders et al., 2013). In addition, the effects of current treatments for psoriasis are limited (Torres and Puig, 2018). Thus, further research on the pathophysiology caused by gut microbiota dysbiosis in psoriasis patients, which may be a breakthrough for pathological mechanism research, is urgently required.

Gut microbiota alterations have been observed in psoriatic patients and psoriatic arthritis patients (Fry and Baker, 2007; Scher et al., 2015; Chen et al., 2018; Hidalgo-Cantabrana et al., 2019; Shapiro et al., 2019). It was also found that gut microbiota dysregulation induced by vancomycin and polymyxin exacerbated psoriasis in an imiquimod (IMQ)-induced mouse model (Zanvit et al., 2015). It has been reported that antibiotic therapy can alleviate the thickness of lesions and reduce the proportion of Th17 cells, γδT cells and IL-17 in an imiquimod-induced psoriasis mouse model (Zákostelská et al., 2016). A predisposition for intestinal bowel diseases among psoriasis patients supports the gut-skin axis theory (Kimball et al., 2014). All of these studies identified the microbiota composition using 16S rRNA sequencing. This method has limitations such as failure to determine most microbes at the strain and species level and the potential for amplification bias (Konstantinidis and Tiedje, 2007; Pinto and Raskin, 2012). In contrast, compared with 16S rRNA sequencing, metagenome sequencing can provide a higher resolution of taxonomic profiles with functional classification of the microbiome (Lee et al., 2018). Nonetheless, few studies on the relationship of the gut microbiome and psoriasis using metagenome sequencing analysis have been conducted.

In this study, to further investigate the intestinal microbiota composition, gene functions and relative metabolites predicted from the microbial composition of psoriasis patients, the profile and composition of the intestinal microbiomes from stool samples of psoriasis patients and healthy controls were compared using metagenomics sequencing analysis. Investigation of the definite role of intestinal microbiome dysbiosis in the immunopathogenesis of inflammatory diseases may boost our realization of psoriasis aetiology and pathological mechanisms and promote targeted and novel predictions and treatments.

## Materials and Methods

### Participants

Patients with psoriasis (n=30) were identified and collected from the Beijing Hospital of Traditional Chinese Medicine (**Table 1**). The control group (n=15) comprised age-, sex- and body mass index (BMI)-matched healthy donors. Patients and healthy donors who received antibiotics, probiotics, glucocorticoids, or immunosuppression during the 6 previous months were not included. People with BMI ≥ 35 or ≤ 18, and those who have been suffering from diseases of the digestive system, immune system and other severe illnesses, were excluded. They have no especially dietary habit and belong to the same ethnic’s population (Chinese). According to principle of statistics, the different sample sizes of two groups will not affect the correctness of statistical inference (Pearce, 2016). The study was approved by the Ethical Committee of the Beijing Hospital of Traditional Chinese Medicine and conformed with the Declaration of Helsinki. All study subjects signed informed consent forms. The questionnaires containing personal and clinical information were completed by the patients.

**Table 1:** Characteristics of the psoriasis patients and healthy individuals.

	Psoriasis	Healthy
Male/female	22/8	11/4
Age (years)*	34 (14)	32 (12)
BMI	24.84 ± 3.73	24.15 ± 2.98
Psoriasis Area Severity Index (PASI)	10.98 ± 3.86	
Types of patients		
Psoriasis vulgaris	30	
Plaque	15	
Guttate	15	
Psoriatic arthritis	0	
Pustulosis of the palms and soles	0	
Erythrodermic psoriasis	0	

*median (mid-quartile range).

### Sample Collection

Stool samples were collected in a specimen collection kit and stored at -20°C immediately after defecation and then at -80°C before further manipulation in the laboratory.

### DNA Extraction and Metagenomic Analysis

DNA from stool samples was extracted using the QIAamp PowerFecal Pro DNA Kit according to the manufacturers’ instructions. DNA was resolved in 10mM Tris-cl buffer and stored at -20°C and at -80°C for short-term and long-term use, respectively. Total DNA quality was measured by using a spectrophotometer with 1% agarose gel electrophoresis. DNA was fragmented to an average size of approximately 350 bp using KAPA HyperPlus PCR-free for library construction. Subsequently, we performed metagenomic sequencing on the Illumina HiSeq platform following the manufacturer’s instructions. The quality control of all raw metagenomic sequencing data was performed using MOCAT2 software. The raw sequence reads were trimmed by SolexaQA package with a length shorter than 30 bp and a quality score lower than 20. The quality control standards are: A260/280 between 1.8-2.0, A260/280>1.5. To remove contaminated reads, the filtered reads were compared to human genome using SOAPaligner, and clean raw reads were obtained. To obtain contigs for the following annotation and prediction, we used SOAPdenovo software to assemble the clean raw reads. Afterwards, the scaftigs from each sample were predicted using MetaGeneMark.

### Bioinformatics and Statistical Analysis

The relative abundances of different level of taxonomic assignment were predicted using MetaPhlAn (metagenomic phylogenetic analysis) 2.0, which includes an expanded set of ~1 million markers from >7,500 species (Truong et al., 2015). We performed all statistical analyses using R software unless otherwise specified. The Shannon indices were used to calculate the α-diversity of the samples. The β-diversity was estimated by taxon-based Bray-Curtis distance *via* the R package vegan, and compositional differences between psoriasis and non-psoriasis samples (β-diversity) were represented in principal coordinates analysis (PCoA) plots using the R package (Bray and Curtis, 1957; VEGAN, 2003; Dray and Dufour, 2007). The nonparametric Kruskal-Wallis test was used because some of the variables analysed lacked homogeneous variances or were not normally distributed. ANCOM analyses and permutational analysis of variance (PERMANOVA) were also performed (Mandal et al., 2015).

Linear discriminant analysis (LDA) effect size (LEfSe) was applied to identify differences in the relative abundances of genera of gut microbiota between psoriasis and healthy volunteers (Segata et al., 2011). LDA values > 2.0 with a *P* value < 0.05 were considered significantly enriched.

### Microbial Gene Function Prediction and Metabolite Analysis

COG and KEGG pathway annotations were obtained using the DIAMOND database (Version 0.7.9.58) *via* BLASTP. To determine gene functions that discriminate bacterial compositions between psoriatic patients and healthy donors, the KEGG enrichment pathways were analysed with Generally Applicable Gene-set Enrichment for Pathway Analysis (GAGE) (Luo et al., 2009). The differential expressed bacteria genes were identified by comparing relative abundances between two groups using Wilcoxon test, and *P* values were adjusted for multiple testing with a Benjamini-Hochberg false discovery rate correction. Adjusted P value < 0.05 were considered significantly differential on gene expression.

The metabolite potential was estimated by the relative abundance of the corresponding species; values were simply added or subtracted if the species produced or consumed, respectively. The production or consumption relationships between metabolites and species were curated from the literature of Sung J et al. and Cecilia Noecker et al. (Noecker et al., 2016; Sung et al., 2017). Heatmaps were generated to represent potential metabolic differences in the gut microbiome between the 2 groups.

## Results

### Microbial Diversity

**Figure 1:**
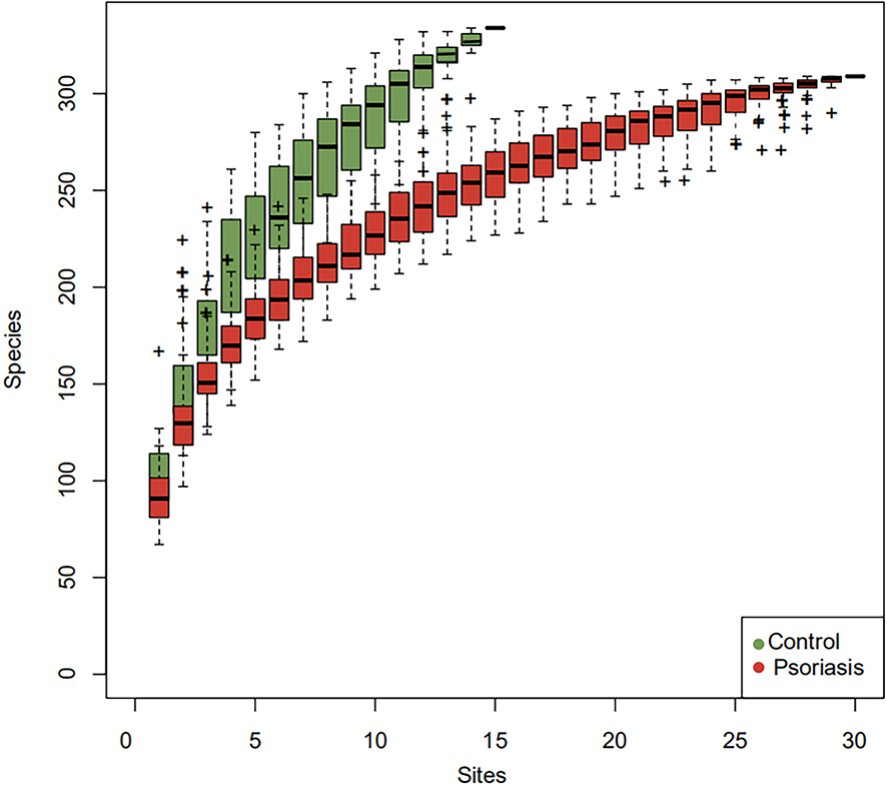
Species accumulation curves. The species accumulation curve of each group tended to plateau, indicating that sample biodiversity was adequately covered with the applied sequencing depth.

**Figure 2:**
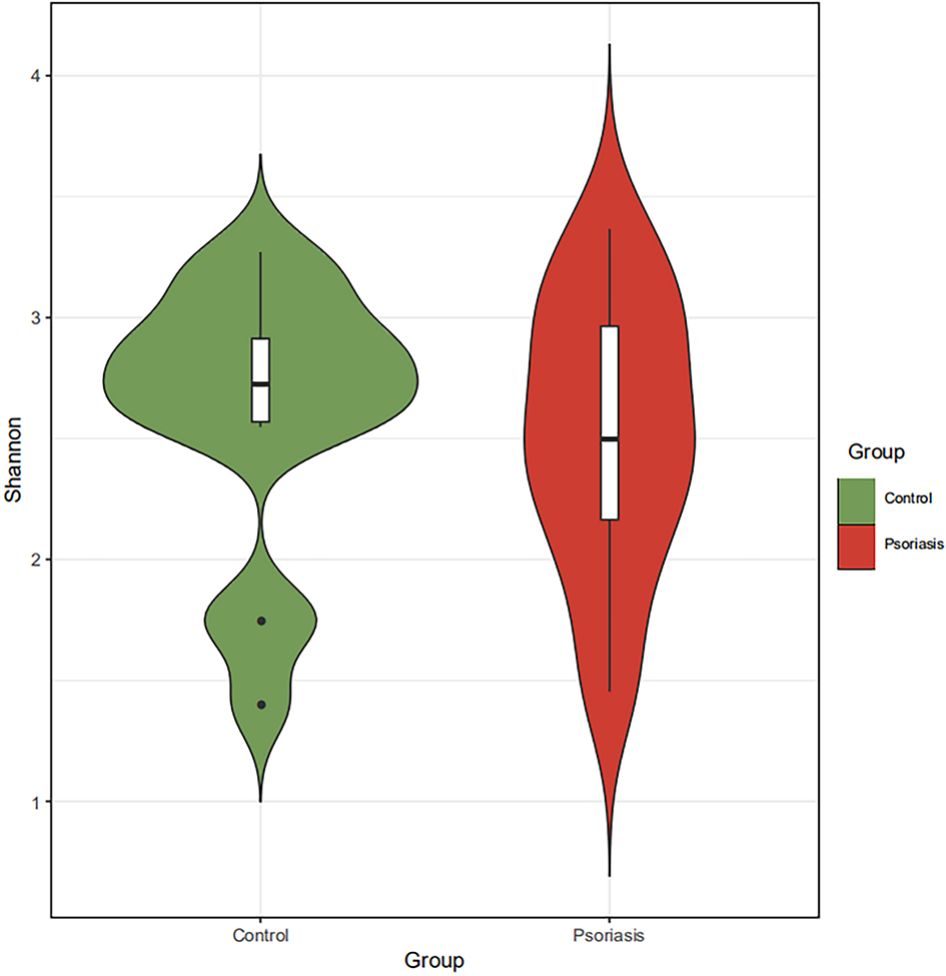
α-Diversity based on Shannon indices displayed no significant differences in the intestinal microbiota between the two groups.

**Figure 3:**
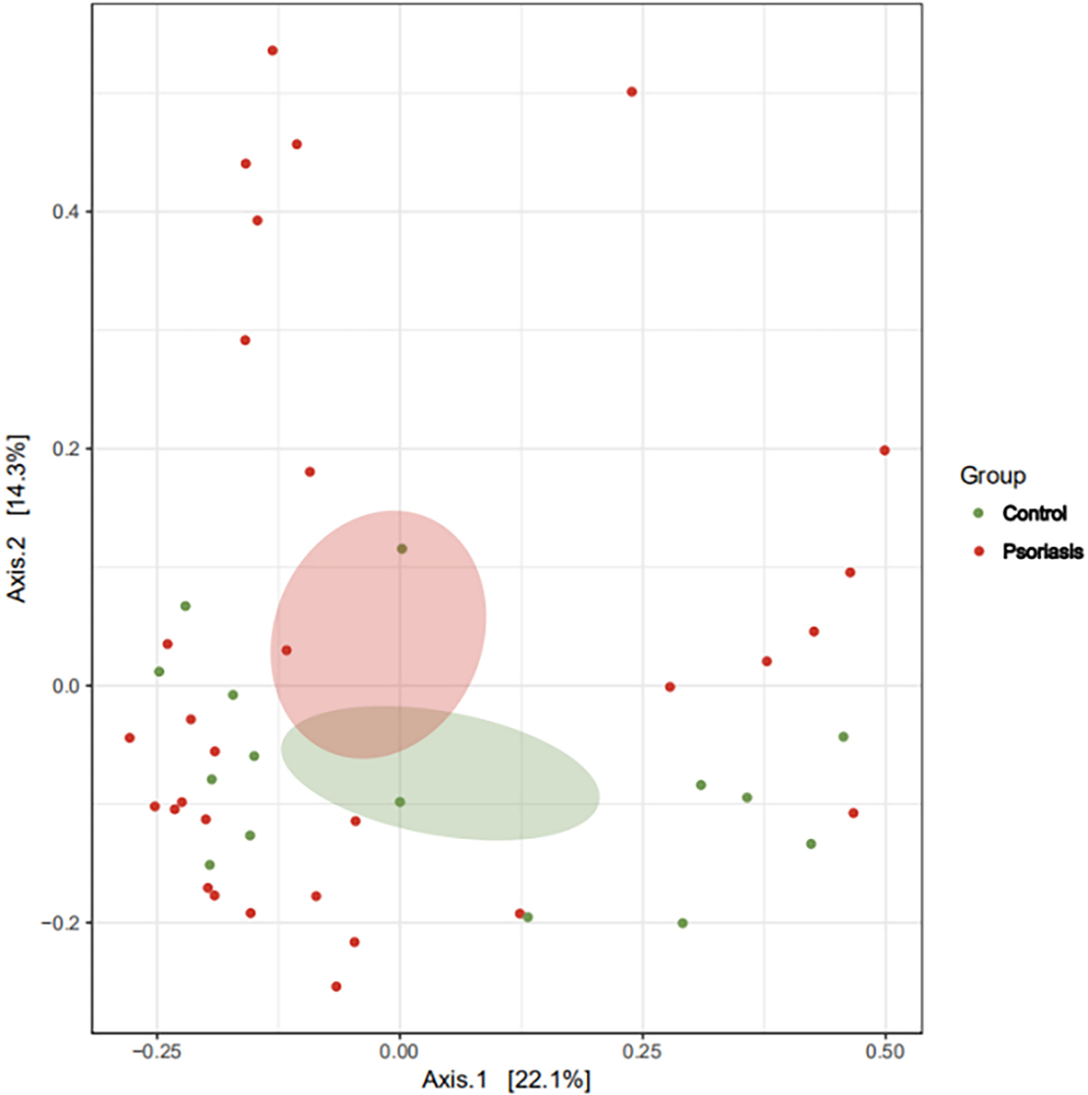
β-Diversity represented as a two-dimensional PCoA showed no differential clustering between the two groups. (The red dots showed the samples from psoriasis patients, whereas the green dots showed the samples from healthy individuals. The closer the dots in one group, the more similar in gut microbiota. The gut microbiota compositions are indicated with red and green circles, respectively. The smaller the overlap of two circles, the more different in the gut microbiota compositions of two groups).

The species accumulation curve of each group tended to plateau, indicating that sample biodiversity was adequately covered with the applied sequencing depth (**Figure 1**). The α-diversity of the gut microbiota (Shannon index) in the healthy group was higher than that in the psoriasis group, but there were no significant differences between the two groups (**Figure 2**). Moreover, the β-diversity analyses showed through PCoA demonstrated a separate tendency, but no significant clustering of non-psoriasis controls and patients was observed based on Bray-Curtis dissimilarity, indicating that the structural diversity of the intestinal microbiota did not differ between the two groups (**Figure 3**).

### Demographics of the Study Population

Forty-five faecal samples were collected: 30 (60%) samples were collected from patients suffering from psoriasis, and 15 samples were collected from healthy age-, sex-, BMI-matched individuals. The demographic characteristics details of the two groups are presented in **Table 1**.

### Altered Gut Microbiota Composition in Psoriasis Patients

**Figure 4:**
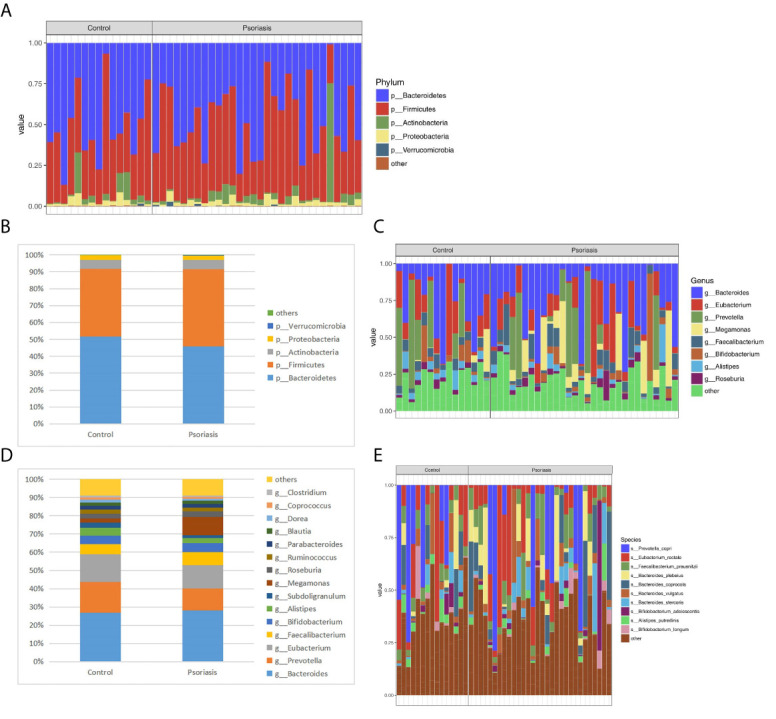
Relative abundance (%) of the intestinal microbiota determined at the phylum **(A, B)** genus **(C, D)** and species **(E)** levels. The columns represent individuals in the psoriasis or control group **(A, C, E)**.

Five dominant phyla based on different relative abundances between psoriasis patients and controls were identified. The phyla *Actinobacteria*, *Firmicutes*, and *Verrucomicrobia* were increased, while *Bacteroidetes* and *Proteobacteria* were reduced in the psoriatic patients compared with the controls (**Figure 4A**, **B**). Moreover, at the genus level, higher proportions of *Faecalibacterium, Bacteroides*, *Bifidobacterium, Megamonas* and *Roseburia* and lower proportions of *Prevotella, Alistipes* and *Eubacterium* were identified in psoriatic patients (**Figures 4C**, **D**). The abundance of *Faecalibacterium prausnitzii* was higher in psoriasis patients than in controls (**Figure 4E**). It has been reported that *Faecalibacterium* and *Prevotella* are associated with anti-inflammatory properties (Shapiro et al., 2019).

Wilcoxon rank-sum permutation tests were performed to further compare the significant differences in the gut microbiota compositions between the 2 groups. There was a significant decrease in the phylum *Euryarchaeota* in the psoriasis group (0.031 vs 0.003, *P* < 0.05). Decreases in the families *Oxalobacteraceae, Porphyromonadaceae, Pasteurellaceae, Rikenellaceae, Sphingobacteriaceae* and *Comamonadaceae* were observed in the psoriasis patients (**Table 2A**). Nine genera, *Butyricimonas, Oxalobacter, Actinobacillus, Odoribacter, Anaerotruncus, Alistipes, Pseudoflavonifractor, Pedobacter*, and *Comamonas*, were significantly more abundant in the healthy control individuals than in the psoriasis patients (**Table 2B**).

**Table 2:** Taxonomic differences between the psoriasis patients and controls at the family **(A)** and genus **(B)** levels.

	**Control**		**Psoriasis**		**W**		***P* value**			***P*.adj**
(A)								
f:Oxalobacteraceae	0.04218		0.00441		307		0.00701			0.50608
f:Porphyromonadaceae	2.99224		2.31140		327		0.01332			0.50608
f:Pasteurellaceae	0.23932		0.06072		311		0.03518			0.54833
f:Rikenellaceae	4.49669		2.81931		309		0.04431			0.54833
f:Sphingobacteriaceae	0.00253		0		255		0.04671			0.54833
f:Comamonadaceae	0.00060		0		255		0.04671			0.54833
(B)									
g:Butyricimonas	0.01147		0		285		0.00371			0.46273
g:Oxalobacter	0.04218		0.00442		307		0.00701			0.46273
g:Actinobacillus	0.00279		0		270		0.01323			0.58210
g:Odoribacter	0.57391		0.17524		317.5		0.02040			0.59475
g:Anaerotruncus	0.01934		0.00789		306.5		0.02997			0.59475
g:Alistipes	4.49669		2.81931		309		0.04431			0.59475
g:Pseudoflavonifractor	0.00011		0		255		0.04671			0.59475
g:Pedobacter	0.00253		0		255		0.04671			0.59475
g:Comamonas	0.00060		0		255		0.04671			0.59475

(1) W indicates the statistical value of the Wilcoxon rank sum test. (2) P.adj indicates the P values corrected by multiple testing.

**Figure 5:**
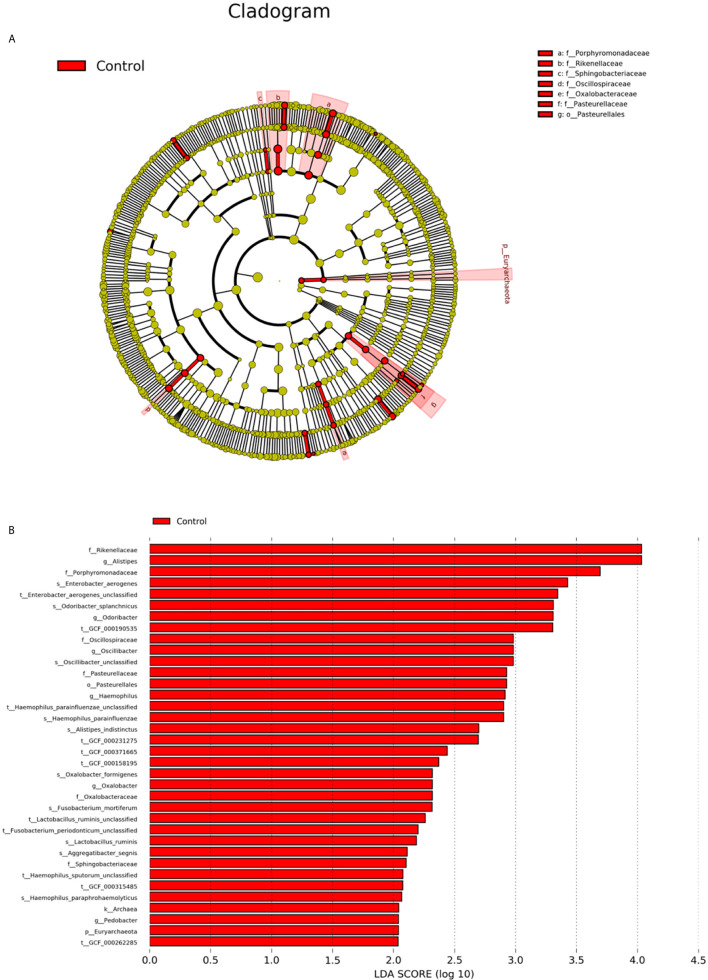
Intestinal microbiota profiles in psoriasis patients. **(A)** Cladogram generated from the LEfSe analysis indicating the phylogenetic distribution of the microbiota of psoriasis and control groups from phylum to genus. **(B)** Histogram of LDA scores to identify differentially abundant bacteria between patients and controls (LDA score > 2.0).

LEfSe was used to further determine the specific significantly different bacterial taxa between the two cohorts (**Figure 5A**). Several genera, including *Oscillospiraceae, Oxalobacter, Haemophilus, Odoribacter, Alistipes*, and *Pedobacter*, were significantly enriched in the faeces of controls. The identified taxa were highlighted on the cladogram to indicate significant differences in phylogenetic distributions along with their LDA scores (**Figure 5B**). These results represented a notable gut microbiota alteration between the 2 groups.

### Gene Functions and Metabolic Variation Analysis in Psoriasis Patients

**Figure 6:**
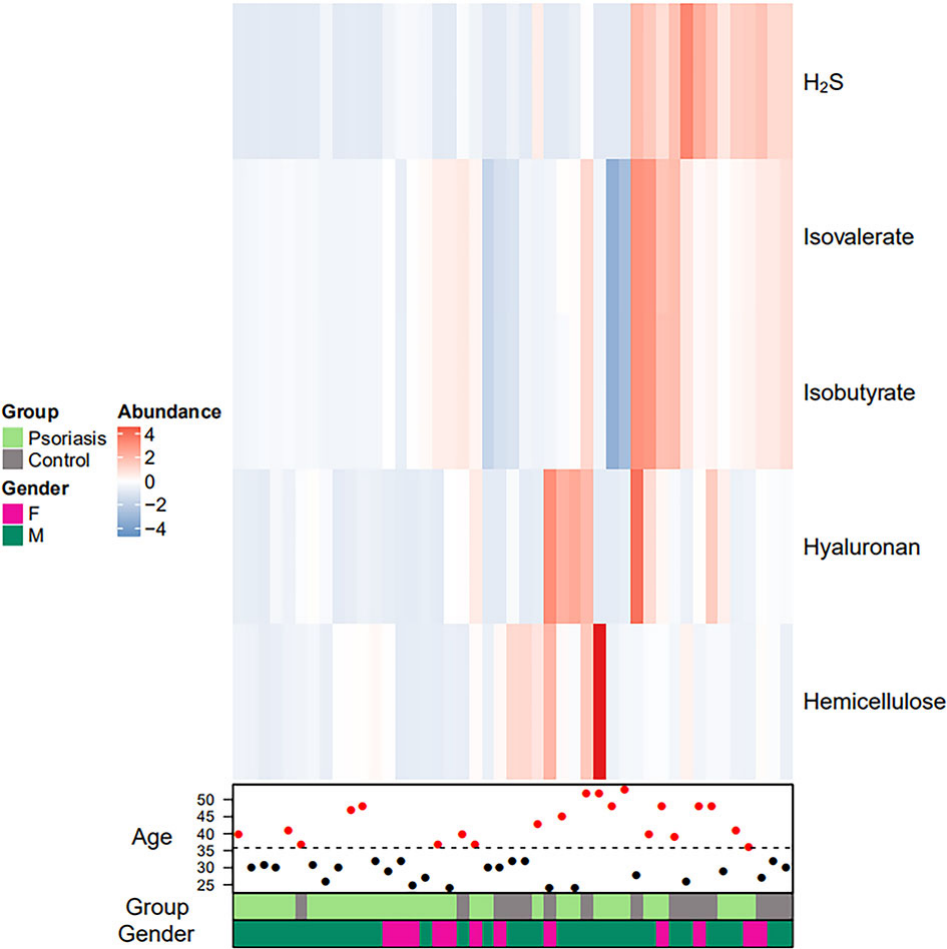
Faecal metabolic heatmap of samples from psoriasis patients. Hierarchical clustering showing the 5 significantly differentially abundant metabolites between the 2 groups (increased and decreased levels of metabolites are indicated by red and blue, respectively). The lines indicate specific metabolites, and the columns represent the individuals.

A total of 134 significantly different functional COGs between patients and controls were predicted (**Table S1**). Fifteen KEGG pathways (including LPS biosynthesis, WNT signaling pathway, apoptosis, bacterial secretion system, phosphotransferase system, and others) were significantly enriched in the psoriasis patient group (**Table 3**). Heatmaps were generated to represent the potential metabolic differences in the gut microbiome between the psoriasis and control groups. We found that 5 metabolites, H_2_S, isovalerate, isobutyrate, hyaluronan and hemicellulose, were significantly differentially abundant between the two cohorts (**Table 4**, **Figure 6**). **Figure 6** shows that the colour of the patient individuals was different from that of the control subjects, which indicated the metabolic differences between the two groups.

**Table 3A:** Upregulated KEGG pathways in psoriasis patients versus healthy subjects.

	stat.mean	*P* value	q value
ko03010 Ribosome	3.03693	0.00166	0.35816
ko03070 Bacterial secretion system	2.62966	0.00475	0.51317
ko02060 Phosphotransferase system (PTS)	2.55145	0.00601	0.64931
ko00051 Fructose and mannose metabolism	2.37617	0.00934	0.67238
ko00052 Galactose metabolism	2.30564	0.01156	0.79040
ko00540 Lipopolysaccharide biosynthesis	2.17960	0.01653	0.79040
ko00290 Valine, leucine and isoleucine biosynthesis	2.10341	0.02329	0.79040
ko00260 Glycine, serine and threonine metabolism	1.67555	0.04828	0.79040

q values indicate the P values corrected with Benjamini and Hochberg false discovery rates.

**Table 3B:** Downregulated KEGG pathways in psoriasis patients versus healthy subjects.

	stat.mean	*P* value	q value
ko02020 Two-component system	-2.57178	0.00516	0.858141
ko04113 Meiosis – yeast	-2.14496	0.02293	0.85814
ko00920 Sulfur metabolism	-1.77403	0.03937	0.85814
ko04114 Oocyte meiosis	-1.84977	0.04057	0.85814
ko04310 WNT signaling pathway	-1.78589	0.04374	0.85814
ko04210 Apoptosis	-1.75035	0.04629	0.85814
ko02010 ABC transporters	-1.68208	0.04651	0.85814

**Table 4:** Dysregulated metabolites in psoriasis patients.

	Control	Psoriasis	W	*P* value	*P*.adj
H_2_S	0.72766	0.22757	346.5	0.00327	0.22888
Hyaluronan	0.83800	0.35367	323	0.01886	0.42476
Hemicellulose	10.65156	7.37906	308	0.04613	0.42476
Isobutyrate	3.57356	0.83686	307	0.04893	0.42476
Isovalerate	3.57504	0.85941	307	0.04893	0.42476

## Discussion

Evidence linking intestinal microbiota dysbiosis to chronic inflammation and immune system-mediated diseases has been expanding. This study is the first to elucidate gut bacterial changes in patients with psoriasis by metagenomic gene profiling analysis, not 16S rRNA sequencing analysis which has limitations but has been used in most studies. For example, contributions to the metagenome by organisms such as bacteria cannot be predicted by using 16S rRNA primers (Shapiro et al., 2019). Furthermore, in this study, the target gene pathways (KEGG and COG) and metabolic function of the microbiome in patients with psoriasis compared with control subjects were also detected, which are beneficial for in depth explanation of the pathogenesis of psoriasis.

Our results indicated the gut microbiota of psoriasis patients displayed an alteration in microbial taxa distribution, but no significant difference in microbial diversity. The psoriatic patients recruited showed a microbiota profile characterized by increased proportions of the phyla *Actinobacteria* and *Firmicutes* and genera *Faecalibacterium, Bacteroides*, *Bifidobacterium* and *Megamonas* and a reduction in the phyla *Bacteroidetes, Euryarchaeota* and *Proteobacteria* and genera *Prevotella, Alistipes* and *Eubacterium*, which seems to be in accordance with results reported in other studies (Scher et al., 2015; Codoñer et al., 2018; Hidalgo-Cantabrana et al., 2019; Shapiro et al., 2019). Functional analysis suggested that 134 COGs were predicted, and 15 KEGG pathways, including LPS biosynthesis, WNT signaling pathway, apoptosis, bacterial secretion system, and phosphotransferase system, were significantly enriched in psoriasis patients. Five metabolites (H_2_S, isovalerate, isobutyrate, hyaluronan, and hemicellulose) were significantly downregulated in the psoriatic cohort.

The pathogenesis of psoriasis could be related to immune and inflammatory response due to the dysfunction of medium- and short-chain fatty acids and the compromised colonic mucosal integrity and gut inflammation (Yegorov et al., 2020). Based on these results, the *Firmicutes/Bacteroidetes (F/B)* ratio increased, and the abundance of *Bacteroides* and *Prevotella* was deregulated in the psoriasis group, which seems to be in accordance with results reported in other studies (Visser et al., 2019; Dei-Cas, et al., 2020; Sikora et al., 2020). It has been reported that a high *F/B* ratio and a deregulated abundance of *Bacteroides* and *Prevotella* alter the production of medium- and short-chain fatty acids, such as acetate and butyrate, which are essential in maintaining colonic mucosal integrity (Corrêa-Oliveira et al., 2016; Perry et al., 2016; Komaroff, 2017; Christensen et al., 2018). The compromised integrity of the mucus barrier affects gut immune homeostasis and antigen presentation, resulting in immune responses and chronic inflammation (Bischoff et al., 2014; Christensen et al., 2018). *Bacteroides* produce polysaccharide A, which activates regulatory T cells and plays an immunomodulatory role in the gut (Mosca et al., 2016). After the addition of arabinoxylans, an elevation in caecal *Prevotella* was detected; *Prevotella* can enhance intestinal barrier function and decrease caecal inflammatory markers (Neyrinck et al., 2011; Neyrinck et al., 2012; Hong et al., 2016). Thus, novel therapeutic approaches that lower the *F/B* ratio and the abundance of *Bacteroides*, as well higher the abundance of *Prevotella* may be beneficial for psoriasis.

Except for immune and inflammatory response, psoriasis aetiology and pathological mechanisms may be also related to dysfunction of gut-brain axis and brain-skin axis. The abundance of *Faecalibacterium prausnitzii* was higher in psoriasis patients than in controls. *F. prausnitzii* has been observed to be decreased in patients with major depressive diseases, RA, metabolic syndrome, Crohn’s disease and obesity (Sokol et al., 2008; Stoll et al., 2014; Jiang et al., 2015; Remely et al., 2016) and increased in children with atopic dermatitis (Song et al., 2016). It has been reported that psoriasis, associated with both a physical and a psychological burden, is closely related to chronic stress (i.e., depression and anxiety) *via* the hypothalamic-pituitary-adrenal (HPA) axis which secretes neuroendocrine mediators and triggers skin inflammation in psoriasis (Chen and Lyga, 2014; Alexopoulos and Chrousos, 2016; Mueller et al., 2017; Visser et al., 2019). In addition, gut-brain axis dysfunction may be associated with psoriasis and psoriatic arthritis (Lu and He, 2015; Sikora et al., 2020), and the altered gut microbiota in patients with psoriatic arthritis resembles dysbiosis in inflammatory bowel disease (Scher et al., 2015). Furthermore, *F. prausnitzii* has effects on cytokine production and can produce butyrate, which inhibits the NF-κB pathway, inhibiting the inflammatory response (Sokol et al., 2008; Jiang et al., 2015; Hiippala et al., 2018). The relative abundances of *Alistipes* and *Parabacteroides* were reduced in the psoriasis group. This result is consistent with studies of psoriasis (Scher et al., 2015; Hidalgo-Cantabrana et al., 2019) and Crohn’s disease (Willing et al., 2010). The abundance of members of the genus *Sutterella* were also found to increase. *Sutterella* has a potential role in immune regulation and pro-inflammatory properties (Hiippala et al., 2016). Therefore, therapies that can lower the abundance of *F. prausnitzii* and *Sutterella*, and higher the abundance of *Alistipes* and *Parabacteroides* may be effective for treating psoriasis. Further research of the role of other identified genera in psoriasis is needed in the future.

A total of 15 KEGG pathways were significantly enriched in the patients with psoriasis, including LPS biosynthesis, WNT signaling pathway, bacterial secretion system, phosphotransferase system, and apoptosis, which are relevant to inflammatory response and apoptosis. LPS has been epidemiologically relevant to psoriasis and may maintain chronic inflammation (Shapiro et al., 2007). This inflammagen will activate the production of inflammatory chemokines and cytokines, stimulating the innate and adaptive immune systems (Kell and Pretorius, 2015; Potgieter et al., 2015; Kell and Pretorius, 2018). In addition, the WNT signaling pathway, which is involved in skin inflammation in psoriasis pathogenesis, was enriched in psoriatic patients and psoriatic arthritis patients (Dolcino et al., 2015; Wang et al., 2017). It has been reported that IL-36γ promotes inflammation and suppresses the differentiation of keratinocytes in psoriasis *via* the WNT signaling pathway (Wang et al., 2017). Moreover, the WNT signaling pathway has been involved in synovial inflammation (Miao et al., 2013). Apoptosis plays a key role in the pathogenesis of psoriasis (Chimenti et al., 2018; Hugh and Weinberg, 2018). T cells cause chronic inflammation and apoptosis *via* cytokines in psoriasis (Hugh and Weinberg, 2018). It has been reported that topical sunitinib ointment can suppress the proliferation and apoptosis of keratinocytes and reduce psoriasis-like inflammation (Kuang et al., 2018). The associations of other enriched pathways and the pathogenesis of psoriasis may be the direction of further research.

Heat map and hierarchical clustering showed the 5 metabolites (H_2_S, isovalerate, isobutyrate, hyaluronan, and hemicellulose) that were significantly differentially abundant between psoriasis patients and controls, which are related to inflammation and the VEGF signaling pathway. Studies have shown that H_2_S is a novel inflammatory mediator (Bhatia, 2015). H_2_S has been involved in different clinical inflammatory disorders, animal models, and *in vitro* systems (Bhatia, 2015; Castelblanco et al., 2018). H_2_S can alleviate LPS-induced lung inflammation in male rats (Zhang et al., 2016; Ali et al., 2018). Remarkable elevation of 3-OH-isovalerate was confirmed in 6 patients with biotinidase deficiency, all of whom presented with some extent of neurological abnormalities and dermatological lesions, and one of them presented with generalized pustular psoriasis (Yang et al., 2003). It has been suggested that butyrate is an intermediate in the anaerobic degradation of isobutyrate (Tholozan et al., 1988). The reduced generation of butyrate in psoriasis patients may compromise the integrity of the intestinal epithelial barrier, resulting in immune responses and chronic inflammation (Bischoff et al., 2014; Christensen et al., 2018; Shapiro et al., 2019). It has been reported that hyaluronan can act as a pro-inflammatory cytokine and induce inflammatory processes in psoriatic arthritis (Hellman et al., 2019). The VEGF signaling pathway plays an important role in psoriasis, and modified hemicellulose suppresses VEGF-induced angiogenesis (Malecic and Young, 2016; Zhu et al., 2017). Chronic inflammation is relevant to alterations of the intestinal microbiota that can be modified with hemicellulose (Konkol et al., 2019).

Lower microbial diversity was detected in some studies (Scher et al., 2015; Hidalgo-Cantabrana et al., 2019), while our results were on their opposite and were consistent with the research of Codoner et al. and Tan et al. (Codoñer et al., 2018; Tan et al., 2018). Difference of those results could be due to the different algorithms used and indices used in the studies, since most studies performed 16S rRNA sequencing and considered the number of OTUs and their relative proportion.

The potential limitations of the study are as follows: (1) The sample size was too small to identify the impact of the length, severity, comorbidities and treatments of psoriasis on the microbiome. (2) The absence of stool supernatant and gut epithelium samples might be beneficial for further research. (3) The microbial metabolite profiling was inferred base on the DNA and the abundance of gut microbiota, not by metabonomics. The actual microbial metabolite profiling may be different from it in the processes of DNA transcription and translation. (4) The gut microbiota profile, genetic functions and relative metabolites of psoriasis patients were performed using metagenomics sequencing. However, the sample marker gene database may include most of the species-and strain-level information, not 100% of all existing species or organisms. (5) The dietary habits have not been screened with specific questionnaire, which may potentially influence the gut microbiota of subjects.

## Conclusion

The demonstrated alterations in microbiome composition and corresponding gene function and metabolic dysregulation likely impact the regulation of inflammation and immune responses, the VEGF signaling pathway, apoptosis, gut-brain axis and brain-skin axis that lead to psoriasis. Understanding the association between the intestinal microbiome and psoriasis pathogenesis will be beneficial for the development of novel predictions and therapeutic approaches for psoriasis, including microbial administration *via* diet, eradication of antibiotics, addition of probiotics or faecal microbial transplantation as a substitute to shift the microbiome towards a healthy status, as has been suggested for IBD (Quraishi et al., 2017).

## Data Availability Statement

The datasets presented in this study can be found in online repositories. The names of the repository/repositories and accession number(s) can be found below: https://www.ncbi.nlm.nih.gov/sra/PRJNA688472.

## Ethics Statement

The studies involving human participants were reviewed and approved by the Ethical Committee of the Beijing Hospital of Traditional Chinese Medicine. The patients/participants provided their written informed consent to participate in this study.

## Author Contributions

GZ and YT designed the study. FX and YL performed the experiments. XL and XW collected and prepared samples for sequencing. MC performed sequencing and sequencing analysis with technical assistance from CJ. HL and XW performed statistical interpretation and analyses with technical assistance from XG, and SX took primary responsibility for writing the manuscript with English improved by YT and GZ. All authors discussed the results and commented on the manuscript. All authors contributed to the article and approved the submitted version.

## Funding

This work was supported by grants from the National Natural Science Foundation of China (grant number 81673974, 81974572) and the Beijing Municipal Administration of Hospitals Incubating Program (grant number PZ2019024).

## Conflict of Interest

MC was employed by the company Beijing QuantiHealth Technology Co., Ltd.

The remaining authors declare that the research was conducted in the absence of any commercial or financial relationships that could be construed as a potential conflict of interest.

## References

[B1] Alexopoulos A. Chrousos G. P. (2016). Stress-related skin disorders. Rev. Endocr. Metab. Disord. 17 (3), 295–304. 10.1007/s11154-016-9367-y 27368716

[B2] Ali F. F. Abdel-Hamid H. A. Toni N. D. (2018). H_2_S attenuates acute lung inflammation induced by administration of lipopolysaccharide in adult male rats. Gen. Physiol. Biophys. 10.4149/gpb_2018002 29956668

[B3] Bhatia M. (2015). H_2_S and Inflammation: An Overview. Handb. Exp. Pharmacol. 230, 165–180. 10.1007/978-3-319-18144-8_8 26162834

[B4] Bischoff S. C. Barbara G. Buurman W. Ockhuizen T. Schulzke J. D. Serino M.. (2014). Intestinal permeability-a new target for disease prevention and therapy. BMC Gastroenterol. 14:189. 10.1186/s12876-014-0189-7 25407511 PMC4253991

[B5] Bray J. R. Curtis J. T. (1957). An ordination of the upland forest communities of Southern Wisconsin. Eco. Monogr. 27, 325–349. 10.2307/1942268

[B6] Castelblanco M. Lugrin J. Ehirchiou D. Nasi S. Ishii I. So A.. (2018). Hydrogen sulfide inhibits NLRP3 inflammasome activation and reduces cytokine production both in vitro and in a mouse model of inflammation. J. Biol. Chem. 293 (7), 2546–2557. 10.1074/jbc.M117.806869 29279328 PMC5818172

[B7] Chen Y. Lyga J. (2014). Brain-Skin Connection: Stress, Inflammation and Skin Aging. Inflammation Allergy Drug Targets 13 (3), 177–190. 10.2174/1871528113666140522104422 24853682 PMC4082169

[B8] Chen C. C. Chen K. J. Kong M. S. Chang H. J. Huang J. L. (2016). Alterations in the gut microbiotas of children with food sensitization in early life. Pediatr. Allergy Immunol. 27 (3), 254–262. 10.1111/pai.12522 26663491

[B9] Chen Y.-J. Ho H. J. Tseng C.-H. Lai Z.-L. Shieh e.-J. Wu C.-Y. (2018). Intestinal microbiota profiling and predicted metabolic dysregulation in psoriasis patients. Exp. Dermatol. 27 (12), 1336–1343. 10.1111/exd.13786 30238519

[B10] Chimenti M. S. Sunzini F. Fiorucci L. Botti E. Fonti G. L. Conigliaro P.. (2018). Potential role of cytochrome c and tryptase in psoriasis and psoriatic arthritis pathogenesis: focus on resistance to apoptosis and oxidative stress. Front. Immunol. 9, 2363. 10.3389/fimmu.2018.02363 30429845 PMC6220124

[B11] Christensen L. Roager H. M. Astrup A. Hjorth M. F. (2018). Microbial enterotypes in personalized nutrition and obesity management. Am. J. Clin. Nutr. 108 (4), 645–651. 10.1093/ajcn/nqy175 30239555

[B12] Codoñer F. M. Ramírez-Bosca A. Climent E. Carrión-Gutierrez M. Guerrero M. Pérez-Orquín J. M.. (2018). Gut microbial composition in patients with psoriasis. Sci. Rep. 8 (1), 3812. 10.1038/s41598-018-22125-y 29491401 PMC5830498

[B13] Collins S. M. Surette M. Bercik P. (2012). The interplay between the intestinal microbiota and the brain. Nat. Rev. Microbiol. 10 (11), 735–742. 10.1038/nrmicro2876 23000955

[B14] Corrêa-Oliveira R. Fachi J. L. Vieira A. Sato F. T. Vinolo M. A. (2016). Regulation of immune cell function by short-chain fatty acids. Clin. Transl. Immunol. 5 (4), e73. 10.1038/cti.2016.17 PMC4855267 27195116

[B15] de Groot P. F. Belzer C. Aydin Ö. Levin E. Levels J. H. Aalvink S.. (2017). Distinct fecal and oral microbiota composition in human type 1 diabetes, an observational study. PloS One 12 (12), e0188475. 10.1371/journal.pone.0188475 29211757 PMC5718513

[B16] Dei-Cas I. Giliberto F. Luce L. Dopazo H. Penas-Steinhardt A. (2020). Metagenomic analysis of gut microbiota in non-treated plaque psoriasis patients stratified by disease severity: development of a new Psoriasis-Microbiome Index. Sci. Rep. 10 (1), 12754. 10.1038/s41598-020-69537-3 32728075 PMC7391695

[B17] Dolcino M. Ottria A. Barbieri A. Patuzzo G. Tinazzi E. Argentino G.. (2015). Gene expression profiling in peripheral blood cells and synovial membranes of patients with psoriatic arthritis. PloS One 10 (6), e0128262. 10.1371/journal.pone.0128262 26086874 PMC4473102

[B18] Dray S. Dufour A. B. (2007). The ade4 package: Implementing the duality diagram for ecologists. J. Stat. Softw. 22 (4), 1–20. 10.18637/jss.v022.i04

[B19] Edwards C. J. Costenbader K. H. (2014). Epigenetics and the microbiome: developing areas in the understanding of the aetiology of lupus. Lupus 23 (6), 505–506. 10.1177/0961203314531636 24763534

[B20] Fry L. Baker B. S. (2007). Triggering psoriasis: the role of infections and medications. Clin. Dermatol. 25 (6), 606–615. 10.1016/j.clindermatol.2007.08.015 18021899

[B21] Hellman U. Engström-Laurent A. Larsson A. Lindqvist U. (2019). Hyaluronan concentration and molecular mass in psoriatic arthritis: biomarkers of disease severity, resistance to treatment, and outcome. Scand. J. Rheumatol. 48 (4), 284–293. 10.1080/03009742.2019.1577490 31032710

[B22] Hidalgo-Cantabrana C. Gómez J. Delgado S. Requena-López S. Queiro-Silva R. Margolles A.. (2019). Gut microbiota dysbiosis in a cohort of patients with psoriasis. Br. J. Dermatol. 181 (6), 1287–1295. 10.1111/bjd.17931 30920647

[B23] Hiippala K. Kainulainen V. Kalliomaki M. Arkkila P. Satokari R. (2016) Mucosal prevalence and interactions with the epithelium indicate commensalism of Sutterella spp. Front. Microbiol. 7, 1706. 10.3389/fmicb.2016.01706 PMC5080374 27833600

[B24] Hiippala K. Jouhten H. Ronkainen A. Hartikainen A. Kainulainen V. Jalanka J.. (2018). The potential of gut commensals in reinforcing intestinal barrier function and alleviating inflammation. Nutrients 10 (8), E988. 10.3390/nu10080988 30060606 PMC6116138

[B25] Honda K. Littman D. R. (2016). The microbiota in adaptive immune homeostasis and disease. Nature 535 (7610), 75–84. 10.1038/nature18848 27383982

[B26] Hong J. Jia Y. Pan S. Jia L. Li H. Han Z.. (2016). Butyrate alleviates high fat diet-induced obesity through activation of adiponectin-mediated pathway and stimulation of mitochondrial function in the skeletal muscle of mice. Oncotarget 7 (35), 56071–56082. 10.18632/oncotarget.11267 27528227 PMC5302897

[B27] Hugh J. M. Weinberg J. M. (2018). Update on the pathophysiology of psoriasis. Cutis 102 (5S), 6–12. 30566550

[B28] Huttenhower C. Kostic A. D. Xavier R. J. (2014). Inflammatory bowel disease as a model for translating the microbiome. Immunity 40 (6), 843–854. 10.1016/j.immuni.2014.05.013 24950204 PMC4135443

[B29] Jiang H. Ling Z. Zhang Y. Mao H. Ma Z. Yin Y.. (2015). Altered fecal microbiota composition in patients with major depressive disorder. Brain Behav. Immunol. 48, 186–194. 10.1016/j.bbi.2015.03.016 25882912

[B30] Kell D. B. Pretorius E. (2015). On the translocation of bacteria and their lipopolysaccharides between blood and peripheral locations in chronic, inflammatory diseases: the central roles of LPS and LPS-induced cell death. Integr. Biol. (Camb.) 7 (11), 1339–1377. 10.1039/c5ib00158g 26345428

[B31] Kell D. B. Pretorius E. (2018). No effects without causes: the iron dysregulation and dormant microbes hypothesis for chronic, inflammatory diseases. Biol. Rev. Camb. Philos. Soc. 93 (3), 1518–1557. 10.1111/brv.12407 29575574 PMC6055827

[B32] Kimball A. B. Leonardi C. Stahle M. Gulliver W. Chevrier M. Fakharzadeh S.. (2014). Demography, baseline disease characteristics and treatment history of patients with psoriasis enrolled in a multicentre, prospective, disease-based registry (PSOLAR). Br. J. Dermatol. 171 (1), 137–147. 10.1111/bjd.13013 24684204 PMC4232924

[B33] Komaroff A. L. (2017). The microbiome and risk for obesity and diabetes. JAMA 317 (4), 355–356. 10.1001/jama.2016.20099 28006047

[B34] Konkol Y. Keskitalo A. Vuorikoski H. Pietilä S. Elo L. L. Munukka E.. (2019). Chronic nonbacterial prostate inflammation in a rat model is associated with changes of gut microbiota that can be modified with a galactoglucomannan-rich hemicellulose extract in the diet. BJU Int. 123 (5), 899–908. 10.1111/bju.14553 30256506

[B35] Konstantinidis K. T. Tiedje J. M. (2007). Prokaryotic taxonomy and phylogeny in the genomic era: advancements and challenges ahead. Curr. Opin. Microbiol. 10 (5), 504–509. 10.1016/j.mib.2007.08.006 17923431

[B36] Kuang Y.-H. Lu Y. Liu Y.-K. Liao L.-Q. Zhou X.-C. Qin Q.-S.. (2018). Topical Sunitinib ointment alleviates Psoriasis-like inflammation by inhibiting the proliferation and apoptosis of keratinocytes. Eur. J. Pharmacol. 824, 57–63. 10.1016/j.ejphar.2018.01.048 29382533

[B37] Lee M.-J. Kang M.-J. Lee S.-Y. Lee E. Kim K. Won S.. (2018). Perturbations of gut microbiome genes in infants with atopic dermatitis according to feeding type. J. Allergy Clin. Immunol. 141 (4), 1310–1319. 10.1016/j.jaci.2017.11.045 29339259

[B38] Littman D. R. Pamer E. G. (2011). Role of the commensal microbiota in normal and pathogenic host immune responses. Cell Host Microbe 10 (4), 311–323. 10.1016/j.chom.2011.10.004 22018232 PMC3202012

[B39] Lu L. He L. (2015). Gut-brain axis dysfunction may be associated with psoriatic arthritis: comment on the article by Scher et al. Arthritis Rheumatol. 67 (8), 2280. 10.1002/art.39151 25919422

[B40] Luo W. Friedman M. S. Shedden K. Hankenson K. D. Woolf P. J. (2009). GAGE: generally applicable gene set enrichment for pathway analysis. BMC Bioinf. 10, 161. 10.1186/1471-2105-10-161 PMC2696452 19473525

[B41] Luo A. Leach S. T. Barres R. Hesson L. B. Grimm M. C. Simar D. (2017). The microbiota and epigenetic regulation of T helper 17/regulatory T cells: in search of a balanced immune system. Front. Immunol. 8, 417. 10.3389/fimmu.2017.00417 28443096 PMC5385369

[B42] Malecic N. Young H. S. (2016). Novel investigational vascular endothelial growth factor (VEGF) receptor antagonists for psoriasis. Expert Opin. Investig. Drugs 25 (4), 455–462. 10.1517/13543784.2016.1153064 26864055

[B43] Mandal S. Van Treuren W. White R. A. Eggesbø M. Knight R. Peddada S. D. (2015). Analysis of composition of microbiomes: a novel method for studying microbial composition. Microb. Ecol. Health Dis. 26, 27663. 10.3402/mehd.v26.27663 26028277 PMC4450248

[B44] Mangiola F. Ianiro G. Franceschi F. Fagiuoli S. Gasbarrini G. Gasbarrini A. (2016). Gut microbiota in autism and mood disorders. World J. Gastroenterol. 22 (1), 361–368. 10.3748/wjg.v22.i1.361 26755882 PMC4698498

[B45] Miao C.-g. Yang Y.-y. He X. Li X.-f. Huang C. Huang Y.. (2013). Wnt signaling pathway in rheumatoid arthritis, with special emphasis on the different roles in synovial inflammation and bone remodeling. Cell Signal 25 (10), 2069–2078. 10.1016/j.cellsig.2013.04.002 23602936

[B46] Mosca A. Leclerc M. Hugot J. P. (2016). Gut microbiota diversity and human diseases: should we reintroduce key predators in our ecosystem? Front. Microbiol. 7, 455. 10.3389/fmicb.2016.00455 27065999 PMC4815357

[B47] Mueller S. M. Hogg S. Mueller J. McKie S. Itin P. Reinhardt J.. (2017). Functional magnetic resonance imaging in dermatology: The skin, the brain and the invisible. Exp. Dermatol. 26 (10), 845–853. 10.1111/exd.13305 28109199

[B48] Neyrinck A. M. Possemiers S. Druart C. de Wiele T. V. De Backer F. Cani P. D.. (2011). Prebiotic effects of wheat arabinoxylan related to the increase in bifidobacteria, Roseburia and Bacteroides/Prevotella in diet-induced obese mice. PloS One 6, e20944. 10.1371/journal.pone.0020944 21695273 PMC3111466

[B49] Neyrinck A. M. Van Hée V. F. Piront N. De Backer F. Toussaint O. Cani P. D.. (2012). Wheat-derived arabinoxylan oligosaccharides with prebiotic effect increase satietogenic gut peptides and reduce metabolic endotoxemia in diet-induced obese mice. Nutr. Diabetes 2, e28. 10.1038/nutd.2011.24 23154683 PMC3302144

[B50] Noecker C. Eng A. Srinivasan S. Theriot C. M. Young V. B. Jansson J. K.. (2016). Metabolic model-based integration of microbiome taxonomic and metabolomic profiles elucidates mechanistic links between ecological and metabolic variation. mSystems 1 (1), e00013–e00015. 10.1128/mSystems.00013-15 PMC4883586 27239563

[B51] Palm N. W. de Zoete M. R. Flavell R. A. (2015). Immune–microbiota interactions in health and disease. Clin. Immunol. 159 (2), 122–127. 10.1016/j.clim.2015.05.014 26141651 PMC4943041

[B52] Parisi R. Symmons D. P. Griffiths C. E. Ashcroft D. M. Identification and Management of Psoriasis and Associated ComorbidiTy (IMPACT) project team (2013). Global epidemiology of psoriasis: a systemic review of incidence and prevalence. J. Invest. Dermatol. 133 (2), 377–385. 10.1038/jid.2012.339 23014338

[B53] Pearce N. (2016). Analysis of matched case-control studies. BMJ 352, i969. 10.1136/bmj.i969 26916049 PMC4770817

[B54] Penders J. Gerhold K. Stobberingh E. E. Thijs C. Zimmermann K. Lau S.. (2013). Establishment of the intestinal microbiota and its role for atopic dermatitis in early childhood. J. Allergy Clin. Immunol. 132 (3), 601–607.e8. 10.1016/j.jaci.2013.05.043 23900058

[B55] Perry R. J. Peng L. Barry N. A. Cline G. W. Zhang D. Cardone R. L.. (2016). Acetate mediates a microbiome-brain-β-cell axis to promote metabolic syndrome. Nature 534 (7606), 213–217. 10.1038/nature18309 27279214 PMC4922538

[B56] Pinto A. J. Raskin L. (2012). PCR biases distort bacterial and archaeal community structure in pyrosequencing datasets. PloS One 7, e43093. 10.1371/journal.pone.0043093 22905208 PMC3419673

[B57] Planer J. D. Peng Y. Kau A. L. Blanton L. V. Ndao I.M. Tarr P. I.. (2016). Development of the gut microbiota and mucosal IgA responses in twins and gnotobiotic mice. Nature 534 (7606), 263–266. 10.1038/nature17940 27279225 PMC4902178

[B58] Potgieter M. Bester J. Kell D. B. Pretorius E. (2015). The dormant blood microbiome in chronic, inflammatory diseases. FEMS Microbiol. Rev. 39 (4), 567–591. 10.1093/femsre/fuv013 25940667 PMC4487407

[B59] Quraishi M. N. Critchlow T. Bhala N. Sharma N. Iqbal T. (2017). Faecal transplantation for IBD management-pitfalls and promises. Br. Med. Bull. 124 (1), 181–190. 10.1093/bmb/ldx040 29140453

[B60] Remely M. Hippe B. Zanner J. Aumueller E. Brath H. Haslberger A. G. (2016). Gut microbiota of obese, type 2 diabetic individuals is enriched in Faecalibacterium prausinitzii, Allermansia muciniphila and Peptostreoptococcus anaerobius after weight loss. Endocr. Metab. Immune Disord. Drug Targets 16 (2), 99–106. 10.2174/1871530316666160831093813 27577947

[B61] Rosser E. C. Mauri C. (2016). A clinical update on the significance of the gut microbiota in systemic autoimmunity. J. Autoimmun 74, 85–93. 10.1016/j.jaut.2016.06.009 27481556

[B62] Salem I. Ramser A. Isham N. Ghannoum M. A. (2018). The gut microbiome as a major regulator of the gut–skin axis. Front. Microbiol. 9, 1459. 10.3389/fmicb.2018.01459 30042740 PMC6048199

[B63] Scher J. U. Ubeda C. Artacho A. Attur M. Isaac S. Reddy S. M.. (2015). Decreased bacterial diversity characterizes the altered gut microbiota in patients with psoriatic arthritis, resembling dysbiosis in inflammatory bowel disease. Arthritis Rheumatol. 67 (1), 128–139. 10.1002/art.38892 25319745 PMC4280348

[B64] Segata N. Izard J. Waldron L. Gevers D. Miropolsky L. Garrett W. S.. (2011). Metagenomic biomarker discovery and explanation. Genome Biol. 12, R60. 10.1186/gb-2011-12-6-r60 21702898 PMC3218848

[B65] Shamriz O. Mizrahi H. Werbner M. Shoenfeld Y. Avni O. Koren O. (2016). Microbiota at the crossroads of autoimmunity. Autoimmun Rev. 15 (9), 859–869. 10.1016/j.autrev.2016.07.012 27392501

[B66] Shapiro J. Cohen A. D. David M. Hodak E. Chodik G. Viner A.. (2007). The association between psoriasis, diabetes mellitus, and atherosclerosis in Israel: a case-control study. J. Am. Acad. Dermatol. 56 (4), 629–634. 10.1016/j.jaad.2006.09.017 17157411

[B67] Shapiro J. Cohen N. A. Shalev V. Uzan A. Koren O. Maharshak N. (2019). Psoriatic patients have a distinct structural and functional fecal microbiota compared with controls. J. Dermatol. 46 (7), 595–603. 10.1111/1346-8138.14933 31141234

[B68] Sikora M. Stec A. Chrabaszcz M. Knot A. Waskiel-Burnat A. Rakowska A.. (2020). Gut microbiome in psoriasis: an updated review. Pathogens 9 (6), 463. 10.3390/pathogens9060463 PMC7350295 32545459

[B69] Sokol H. Pigneur B. Watterlot L. Lakhdari O. Bermúdez-Humarán L. G. Gratadoux J. J.. (2008). Faecalibacterium prausnitzii is an anti-inflammatory commensal bacterium identified by gut microbiota analysis of Crohn disease patients. Proc. Natl. Acad. Sci. U. S. A. 105 (43), 16731–16736. 10.1073/pnas.0804812105 18936492 PMC2575488

[B70] Sommer F. Backhed F. (2013). The gut microbiota-masters of host development and physiology. Nat. Rev. Microbiol. 11 (4), 227–238. 10.1038/nrmicro2974 23435359

[B71] Song H. Yoo Y. Hwang J. H. Na Y. C. Kim H. S. (2016). Faecalibacterium prausinitzii subspecies-level dysbiosis in the human gut microbiome underlying atopic dermatitis. J. Allergy Clin. Immunol. 137 (3), 852–860. 10.1016/j.jaci.2015.08.021 26431583

[B72] Stockinger B. Omenetti S. (2017). The dichotomous nature of T helper 17 cells. Nat. Rev. Immunol. 17 (9), 535–544. 10.1038/nri.2017.50 28555673

[B73] Stoll M. L. Kumar R. Morrow C. D. Lefkowitz E. J. Cui X. Genin A.. (2014). Altered microbiota associated with abnormal humoral immune response to commensal organisms in entehsitis-related arthritis. Arthritis Res. Ther. 16 (6), 486. 10.1186/s13075-014-0486-0 25434931 PMC4272554

[B74] Sung J. Kim S. Cabatbat J. J. T. Jang S. Jin Y.-S. Jung G. Y.. (2017). Global metabolic interaction network of the human gut microbiota for context-specific community-scale analysis. Nat. Commun. 8, 15393. 10.1038/ncomms15393 28585563 PMC5467172

[B75] Tan L. Zhao S. Zhu W. Wu L. Li J. Shen M.. (2018). The Akkermansia muciniphila is a gut microbiota signature in psoriasis. Exp. Dermatol. 27 (2), 144–149. 10.1111/exd.13463 29130553

[B76] Tholozan J.-L. Samain E. Grivet J.-P. (1988). Isomerization between n-butyrate and isobutyrate in enrichment cultures. FEMS Microbiol. Ecol. 4, 187–191. 10.1111/j.1574-6968.1988.tb02663.x

[B77] Torres T. Puig L. (2018). Apremilast: A Novel Oral Treatment for Psoriasis and Psoriatic Arthritis. Am. J. Clin. Dermatol. 19 (1), 23–32. 10.1007/s40257-017-0302-0 28597182

[B78] Truong D. T. Franzosa E. A. Tickle T. L. Scholz M. Weingart G. Pasolli E.. (2015). MetaPhlAn2 for enhanced metagenomic taxonomic profiling. Nat. Methods 12 (10), 902–903. 10.1038/nmeth.3589 26418763

[B79] Vegan D. P. (2003). A package of R functions for community ecology. J. Veg. Sci. 14, 927–930. 10.1111/j.1654-1103.2003.tb02228.x

[B80] Visser M. J.E. Kell D. B. Pretorius E. (2019). Bacterial dysbiosis and translocation in psoriasis vulgaris. Front. Cell Infect. Microbiol. 4, 7. 10.3389/fcimb.2019.00007 PMC6369634 30778377

[B81] Wang W. Yu X. Wu C. Jin H. (2017). IL-36γ inhibits differentiation and induces inflammation of keratinocyte via Wnt signaling pathway in psoriasis. Int. J. Med. Sci. 14 (10), 1002–1007. 10.7150/ijms.20809 28924372 PMC5599924

[B82] Willing B. P. Dicksved J. Halfvarson J. Andersson A. F. Lucio M. Zheng Z.. (2010). A pyrosequencing study in twins shows that gastrointestinal microbial profiles vary with inflammatory bowel disease phenotypes. Gastroenterology 139 (6), 1844–1854.e1. 10.1053/j.gastro.2010.08.049 20816835

[B83] Yan D. Issa N. Afifi L. Jeon C. Chang H. W. Liao W. (2017). The role of the skin and gut microbiome in psoriatic disease. Curr. Dermatol. Rep. 6 (2), 94–103. 10.1007/s13671-017-0178-5 28804689 PMC5552074

[B84] Yang Y. L. Yamaguchi S. Tagami Y. Zhang Y. H. Xiong H. Hasegawa Y.. (2003). Diagnosis and treatment of biotinidase deficiency-clinical study of six patients. Zhonghua Er Ke Za Zhi 41 (4), 249–251. Chinese. 14754524

[B85] Yegorov S. Babenko D. Kozhakhmetov S. Akhmaltdinova L. Kadyrova I. Nurgozhina A.. (2020). Psoriasis is associated with elevated gut IL-1α and intestinal microbiome alterations. Front. Immunol. 1, 571319. 10.3389/fimmu.2020.571319 PMC7559734 33117362

[B86] Yeoh N. Burton J. P. Suppiah P. Reid G. Stebbings S. (2013). The role of the microbiome in rheumatic diseases. Curr. Rheumatol. Rep. 15 (3), 314. 10.1007/s11926-012-0314-y 23378145

[B87] Zákostelská Z. Málková J. Klimešová K. Rossmann P. Hornová M. Novosádová I.. (2016). Intestinal microbiota promotes psoriasis-like skin inflammation by enhancing Th17 response. PloS One 11 (7), e0159539. 10.1371/journal.pone.0159539 27434104 PMC4951142

[B88] Zanvit P. Konkel J. E. Jiao X. Kasagi S. Zhang D. Wu R.. (2015). Antibiotics in neonatal life increase murine susceptibility to experimental psoriasis. Nat. Commun. 6, 8424. 10.1038/ncomms9424 26416167 PMC4598725

[B89] Zhang H.-X. Liu S.-J. Tang X.-L. Duan G.-L. Ni X. Zhu X.-Y.. (2016). H_2_S Attenuates LPS-Induced Acute Lung Injury by Reducing Oxidative/Nitrative Stress and Inflammation. Cell Physiol. Biochem. 40 (6), 1603–1612. 10.1159/000453210 28006762

[B90] Zhang M. Ma W. Zhang J. He Y. Wang J. (2018). Analysis of gut microbiota profiles and microbe–disease associations in children with autism spectrum disorders in China. Sci. Rep. 8 (1), 13981. 10.1038/s41598-018-32219-2 30228282 PMC6143520

[B91] Zhu X. Okubo A. Igari N. Ninomiya K. Egashira Y. (2017). Modified rice bran hemicellulose inhibits vascular endothelial growth factor-induced angiogenesis in vitro via VEGFR2 and its downstream signaling pathways. Biosci. Microbiota Food Health 36 (2), 45–53. 10.12938/bmfh.16-016 28439487 PMC5395424

